# Proteomic analysis of pulmonary arteries and lung tissues from dogs affected with pulmonary hypertension secondary to degenerative mitral valve disease

**DOI:** 10.1371/journal.pone.0296068

**Published:** 2024-01-05

**Authors:** Siriwan Sakarin, Anudep Rungsipipat, Sittiruk Roytrakul, Janthima Jaresitthikunchai, Narumon Phaonakrop, Sawanya Charoenlappanit, Siriwan Thaisakun, Sirilak Disatian Surachetpong

**Affiliations:** 1 Department of Veterinary Medicine, Faculty of Veterinary Science, Chulalongkorn University, Bangkok, Thailand; 2 Center of Excellence for Companion Animal Cancer, Department of Pathology, Faculty of Veterinary Science, Chulalongkorn University, Bangkok, Thailand; 3 Functional Proteomics Technology Laboratory, National Center for Genetic Engineering and Biotechnology (BIOTEC), National Science and Technology Development Agency, Pathum Thani, Thailand; University of Colorado - Anschutz Medical Campus, UNITED STATES

## Abstract

In dogs with degenerative mitral valve disease (DMVD), pulmonary hypertension (PH) is a common complication characterized by abnormally elevated pulmonary arterial pressure (PAP). Pulmonary arterial remodeling is the histopathological changes of pulmonary artery that has been recognized in PH. The underlying mechanisms that cause this arterial remodeling are poorly understood. This study aimed to perform shotgun proteomics to investigate changes in protein expression in pulmonary arteries and lung tissues of DMVD dogs with PH compared to normal control dogs and DMVD dogs without PH. Tissue samples were collected from the carcasses of 22 small-sized breed dogs and divided into three groups: control (n = 7), DMVD (n = 7) and DMVD+PH groups (n = 8). Differentially expressed proteins were identified, and top three upregulated and downregulated proteins in the pulmonary arteries of DMVD dogs with PH including SIK family kinase 3 (SIK3), Collagen type I alpha 1 chain (COL1A1), Transforming growth factor alpha (TGF-α), Apoptosis associated tyrosine kinase (AATYK), Hepatocyte growth factor activator (HGFA) and Tyrosine-protein phosphatase non-receptor type 13 (PTPN13) were chosen. Results showed that some of the identified proteins may play a role in the pathogenesis of pulmonary arterial remodeling. This study concluded shotgun proteomics has potential as a tool for exploring candidate proteins associated with the pathogenesis of PH secondary to DMVD in dogs.

## Introduction

Degenerative mitral valve disease (DMVD) is the most common acquired cardiac disease in aging small sized breed dogs. It is characterized by thickening of mitral valve, which leads to incomplete valve coaptation and regurgitation from the left ventricle to the left atrium. This results in remodeling of the left atrium and ventricle and can lead to left-sided heart failure [1]. Some dogs may develop pulmonary hypertension (PH) and right-sided heart failure [2]. Pulmonary hypertension, which is a common complication in dogs affected with DMVD, is characterized by an abnormal increase in pulmonary arterial pressure (PAP) and is a poor prognosis indicator for dogs with DMVD [3]. Until recently, there was no specific treatment for dogs with PH secondary to DMVD [4].

Pulmonary arterial remodeling, which is characterized by medial thickening of the pulmonary artery walls, is a common histopathological change found in human patients with PH [5], PH-induced animal models [6] and dogs with PH secondary to DMVD [7]. The etiology of medial thickening in PH is not fully understood [8], but several factors have been suggested to contribute to its development, such as imbalances between vasoconstriction and vasodilation [2], dysregulated cell proliferation and apoptosis [6] and infiltration of inflammatory cells [9].

Several factors have been suggested to be associated with medial thickening in DMVD dogs with PH, such as the serotonin pathway [10], and apoptosis pathway [11]. To gain a more comprehensive understanding of the mechanisms and identify potential targets for therapeutic and diagnostic intervention, it is important to explore other candidate factors or proteins that may be associated with the pathogenesis of medial thickening in DMVD dogs with PH. Mass spectrometry-based proteomics is a suitable technique for this purpose, as it can detect and identify thousands of proteins in biological samples without prior knowledge of the protein of interest. The Liquid Chromatography—Tandem Mass Spectrometry technique (LC-MS/MS), when combined with a database search engine, has been used to identify potential protein candidates associated with diseases [12]. In human medicine, this approach has been used to uncover the mechanisms underlying PH by using several types of specimens including heart, lung tissues and different cell types, such as pulmonary arterial smooth muscle cells (PASMCs) or pulmonary artery endothelial cells (PAECs) from PH human patients and PH-induced animal models [13–16]. Findings from several proteomic studies have summarized those mechanisms such as mitochondrial dysfunction and metabolic shifts, nitric oxide production and responsiveness, coagulation and fibrinolysis, immune and inflammation responses and apoptosis resistance are associated with PH. The information provides valuable insights for further in-depth research [17]. While many proteomic studies have been performed to uncover mechanisms underlying PH in human patients and animal models, no work has been performed on the lung or pulmonary artery proteome in dogs affected with PH secondary to DMVD. The purpose of this study is to investigate changes in protein expression in the pulmonary arteries and lung tissues of DMVD dogs with PH compared to normal control dogs and DMVD dogs without PH.

## Materials and methods

### Animals

The pulmonary artery and lung tissue samples were obtained form 22 older small-sized breed dogs that were donated for necropsy at the Department of Pathology, Faculty of Veterinary Science, Chulalongkorn University, Thailand. Ethical approval is not required because the study was performed in donation cadavers. The medical records of all dogs were reviewed, and the mitral valve degeneration and thickness were examined and measured at necropsy using a vernier caliper. The sample sizes of the groups were determined incrementally based on the tissues available from our prior studies [18]. All samples were divided into three groups: the control group (n = 7), the DMVD group (n = 7) and the DMVD+PH group (n = 8). Dogs with pre-existing cardiovascular diseases other than DMVD, pulmonary diseases, heartworm infection, systemic hypertension, neoplasia, and systemic diseases, such as kidney and liver diseases were not included in the study, as determined by prior medical records and post-mortem examination.

Dogs without any prior cardiorespiratory diseases, and without any signs of mitral valve degeneration or mitral valve thickness less than 2 mm [19] were included to the control group. Dogs with mitral valve nodules and thickness greater than 2 mm were classified as having DMVD [19]. To participate in this study, these dogs had to have been previously diagnosed as DMVD stage C or D with or without PH, as well as evidence of cardiomegaly (vertebral heart score > 10.5) and pulmonary edema as determined by thoracic radiography. Echocardiographic results were used to further classify these dogs into the DMVD or DMVD+PH groups. Dogs with mitral valve thickening and regurgitation, and left-sided heart enlargement, as determined by the ratio of left atrial to aorta dimension (LA/Ao) using the Swedish method ≥ 1.6 and left ventricular internal diameter during diastole normalized with the Allometric scale method (LVIDdN) ≥ 1.7 [20], and without evidence of tricuspid regurgitation (TR) were classified to the DMVD group. DMVD dogs with intermediate to high probability of PH were classified as the DMVD+PH group, if they had TR with peak TR velocity > 3.4 m/s or estimated PAP calculated by using the modified Bernoulli equation (pressure gradient = 4 x peak TR velocity^2^) > 46 mmHg, with or without anatomic structure changes in the ventricles, pulmonary artery, right atrium and caudal vena cava as determined by echocardiography [3, 4]. Dogs with other causes of PH other than DMVD were excluded from the study.

### Sample collection and preparation

In each dog, 1 cm^3^ of peripheral regions of left lung lobe was collected by dissecting the pulmonary artery of the left lung lobe from the lobar to the subsegmental level. These samples were stored in plain tube Eppendorf at -80°C for later assay. Whole dissected pulmonary artery and approximately 100 mg of lung tissue were used for proteomic analysis. Briefly, tissue samples were ground under liquid nitrogen, then diluted with 0.5% sodium dodecyl sulfate (SDS). The samples were centrifuged at 12,000 rpm for 15 minutes, and the supernatants were collected and stored at -20°C for further proteomic analysis.

### Proteomics analysis by LC-MS/MS

The total protein concentration of each tissue sample was determined using the Lowry method with bovine serum albumin (BSA) as the protein standard. Disulfide bonds in the protein samples were reduced by incubating them with 10 mM dithiothreitol (DTT) in 10 mM ammonium bicarbonate for 1 hour at room temperature. The samples were then incubated with 100 mM iodoacetamide (IAA) in 10 mM ammonium bicarbonate in the dark for 1 hour at room temperature to alkylate cysteine residues in the proteins. The protein samples were digested with trypsin (Sequencing Grade, Promega, Germany) at ratio 1:20 (w/w), and tryptic digestion was performed overnight at room temperature. Finally, 0.1% Formic acid (FA) was added to stop enzymatic digestion.

The resulting peptide samples were analyzed using a Liquid Chromatography system (Thermo Scientific Dionex, US) connected to a Hybrid quadrupole Q-Tof impact II™ (Bruker Daltonics) equipped with a Nano-captive spray ion source. Three replicates of each sample were performed. The LC-MS/MS data were analyzed using MaxQuant (version 1.6.6.0) and submitted to a database search against the Uniprot *Canis lupus familiaris* database for protein identification [21]. A Venn diagram was used to illustrate the identified proteins in each group [22]. Proteins that showed at least a 2-fold significant difference between groups (p-value < 0.05) were selected as candidate proteins, including both upregulated and downregulated proteins. The relationship of the differentially expressed proteins with cardiovascular drugs was analyzed using the STITCH database [23]. The MS/MS raw data and analysis files have been deposited in the ProteomeXchange Consortium (http://proteomecentral.proteomexchange.org) via the jPOST partner repository (https://jpostdb.org) with the data set identifier JPST002315 and PXD045240 (preview URL for reviewers: https://repository.jpostdb.org/preview/113963869164fc8225c462f, Access key: 4255).

### Statistical analysis

The data were analyzed using the computer-based software SPSS (version 22, IBM, USA). The normality of the data was tested using the Shapiro-Wilk test, and the normally distributed data were presented as mean and standard deviation (SD). One-way analysis of variance (ANOVA) was used to identify significant differences among the control, DMVD and DMVD+PH groups. Post-hoc analysis was conducted using the Bonferroni test. The differentially expressed proteins between groups were identified using the Independent T-test with a two-tailed approach. The statistical significance was set at p-value of < 0.05.

## Results

### Clinical characteristic of dogs

Twenty-two canine carcasses were included in this study and divided into 3 groups: the control group (n = 7), the DMVD group (n = 7) and the DMVD+PH group (n = 8). The clinical characteristics of the dogs enrolled in this study including age, weight, sex, breed, stage of DMVD and prescribed cardiovascular drugs are summarized in Table 1. The age and weight of dogs were similar across groups, and there was a roughly equal distribution of male and female dogs in each group. According to antemortem diagnostic records, all dogs diagnosed with DMVD displayed signs of congestive heart failure (CHF) and were classified as being stage C or D, according to the ACVIM consensus guidelines for the diagnosis and treatment of myxomatous mitral valve disease in dogs [20]. The most frequently used cardiovascular drugs in this study were angiotensin converting enzyme inhibitor (ACEIs), diuretics, inotropic agents and vasodilators. All dogs diagnosed with DMVD, regardless of the presence or absence of PH, received standard treatment, which included ACEIs, furosemide, and pimobendan. Some of the dogs also received the additional diuretic drugs, such as the combination of amirolide and hydrochlorithiazide (Moduretic®) and spironolactone. Two of the eight dogs in the DMVD+PH group presented with the signs of right-sided heart failure such as ascites, but only one of these dogs was given sildenafil as part of their treatment regimen.

**Table 1:** The clinical characteristics and prescribed cardiovascular drugs of dogs in the control, DMVD and DMVD+PH group.

Parameters	Control (n = 7)	DMVD (n = 7)	DMVD+PH (n = 8)	p-value
Age (year)	11.57 ± 3.36	13.71 ±1.80	13.63 ± 1.60	0.177
Weight (kg)	6.45 ± 2.25	4.91 ± 1.50	6.43 ± 2.81	0.361
Sex				
Male	4 (M = 2, Mc = 2)	3 (M = 1, Mc = 2)	4 (M = 2, Mc = 2)	
Female	3 (F = 1, Fs = 2)	4 (F = 2, Fs = 2)	4 (F = 3, Fs = 1)	
Breed	Shih Tzu (n = 4),	Poodle (n = 4),	Poodle (n = 2),	
	Mixed (n = 2),	Shih Tzu (n = 2),	Chihuahua (n = 2),	
	Pomeranian (n = 1)	Pomeranian (n = 1)	Shih Tzu (n = 1),	
			Pomeranian (n = 1),	
			Schnauzer (n = 1),	
			Yorkshire terrier (n = 1)	
Stage (C/D)	-	4/3	4/4	
Medication	-	ACEIs (n = 7)	ACEIs (n = 8)	
		Furosemide (n = 7)	Furosemide (n = 8)	
		Pimobendan (n = 7)	Pimobendan (n = 8)	
		Spironolactone (n = 3)	Spironolactone (n = 4)	
		amirolide and hydrochlorithiazide (n = 1)	amirolide and hydrochlorithiazide (n = 2)	
			Sildenafil (n = 1)	

Data are reported as mean ± SD.

The significant difference was assessed by the one-way ANOVA at p < 0.05.

The dogs in the control group died from various conditions and diseases that were not related to PH, according to the causes of death. These causes included car accidents as well as postoperative complications, such as peritoneopericardial diaphragmatic hernia, complications from cholecystectomy, pyometra, and gastrointestinal foreign bodies. All of dogs diagnosed with DMVD, both with and without PH, died as a result of cardiorespiratory failure with the exception of one dog in the DMVD+PH group which, was euthanized due to adverse events associated with disease progression and resistance to cardiovascular medications. At necropsy, the control group had no abnormal lesions in the heart and lungs, while the DMVD and DMVD+PH groups showed mitral valve nodularity and thickening, frothy fluid in the trachea and evidence of pulmonary edema from cut surfaces of lung lobes.

### Differential expression of proteins identified by shotgun proteomics

#### Pulmonary arteries

**Fig 1:**
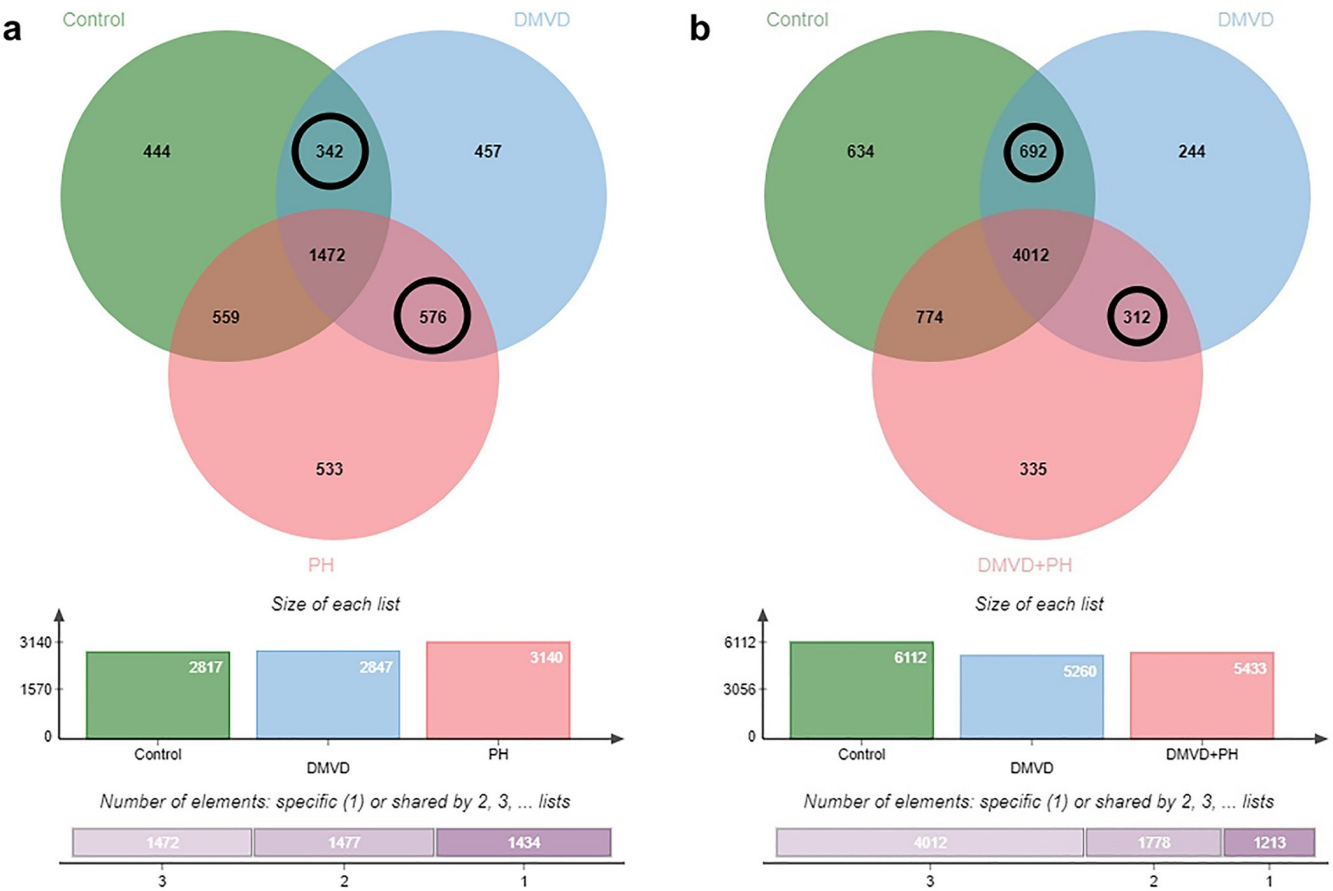
Venn diagram showed the distribution and overlap of identified proteins. Proteins in the pulmonary arteries (a) and lung tissues (b) of dogs in the control, DMVD and DMVD+PH groups. The proteins that commonly found between groups were pointed out as circle. In the pulmonary arteries, 342 proteins were commonly found in the control and DMVD groups and 576 proteins were commonly found in the DMVD and DMVD+PH groups. In the lung tissues, 692 proteins were commonly found in the control and DMVD groups and 312 proteins were commonly found in the DMVD and DMVD+PH groups.

**Fig 2:**
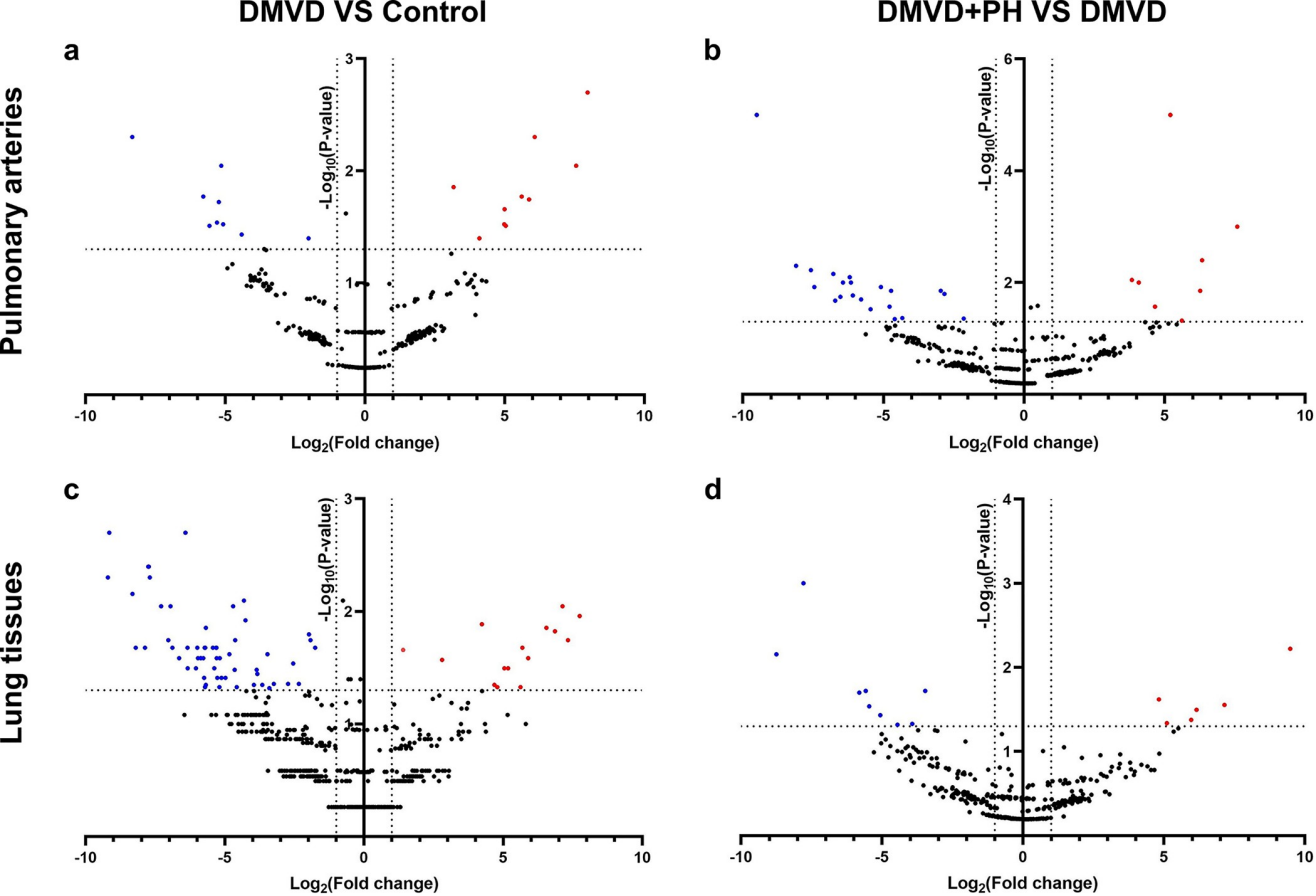
Volcano plot for the identified proteins that were commonly found. Protein in the pulmonary arteries and lung tissues of dogs in the control and DMVD groups (a, c) and in the DMVD and DMVD+PH groups (b, d). Each dot represents an individual protein, red dots were significant upregulated proteins and blue dots were significant downregulated proteins compared between groups. Vertical lines indicate 2-fold difference and horizontal line indicates p-value = 0.05.

The distribution of the identified proteins in the pulmonary arteries of dogs in the control, DMVD and DMVD+PH groups is illustrated in a Venn Diagram (Fig 1A). The total number of identified proteins was 2,817, 2,847 and 3,140 for the control, DMVD and DMVD+PH groups, respectively. The Venn diagram showed that 342 identified proteins were found commonly to be present in both the control and DMVD groups, while 576 identified proteins were common in both the DMVD and DMVD+PH groups (Fig 1A). Among the proteins found to be commonly presented among the group, those with a significant differential abundance (> 2-fold difference, p < 0.05) were identified and illustrated in a Volcano plot (Fig 2). The pulmonary arteries of DMVD dogs compared to normal dogs showed 19 differentially expressed proteins (10 upregulated and 9 downregulated) as illustrated in Fig 2A. On the other hand, the pulmonary arteries of DMVD dogs with PH produced 29 differentially expressed proteins compared to DMVD dogs without PH (8 upregulated and 21 downregulated) (Fig 2B).

In this study, we focused on the top 3 upregulated and downregulated proteins that are believed to be associated with the development of the disease (Table 2). The upregulated proteins in the pulmonary arteries of DMVD dogs compared to the control dogs include Calcium voltage-gated channel auxiliary subunit beta 3 (CACNB3), insulin-like growth factor II (IGF2) and Microtubule associated protein 1B (MAP1B). The downregulated proteins include ADAMTS like proteins 3 (ADAMTSL3), Nucleoside diphosphate kinase (NDPK) and Regulators of G protein signaling 3 (RGS3). Furthermore, in the pulmonary arteries of DMVD dogs with PH compared to the DMVD dogs, the upregulated proteins including SIK family kinase 3 (SIK3), Collagen type I alpha 1 chain (COL1A1) and Transforming growth factor alpha (TGF-α) and the downregulated proteins including Apoptosis associated tyrosine kinase (AATYK), Hepatocyte growth factor activator (HGFA) and Tyrosine-protein phosphatase non-receptor type 13 (PTPN13) were also identified.

**Table 2:** The top 3 upregulated and downregulated proteins in the pulmonary arteries and lung tissues of DMVD dogs compared to the control dogs and DMVD dogs with PH compared to the DMVD dogs.

Group	Pulmonary arteries		Lung tissues	
	Upregulation	Downregulation	Upregulation	Downregulation
**DMVD vs Control groups**	CACNB3	ADAMTSL3	HNMT	TOP1
	IGF2	NDPK	CTNND1	ESRP2
	MAP1B	RGS3	LB1	IFT88
**DMVD+PH vs DMVD groups**	SIK3	AATYK	TLRS	ZEB1
	COL1A1	HGFA	Wnt protein	MCF2L
	TGF-α	PTPN13	UCH	DAPK2

AATYK, Apoptosis associated tyrosine kinase; ADAMTSL3, ADAMTS like proteins 3; CACNB3, Calcium voltage-gated channel auxiliary subunit beta 3; COL1A1, Collagen type I alpha 1 chain; CTNND1, Catenin delta1; DAPK2, Death associated protein kinase 2; ESRP2, Epithelial splicing regulatory protein 2; HGFA, Hepatocyte growth factor activator; HNMT, Histamine N-methyltransferase; IFT88, Intraflagellar transport 88; IGF2, insulin-like growth factor II; LB1, Lamin B1; MAP1B, Microtubule associated protein 1B; MCF2L, MCF2 cell line derived transforming sequence; NDPK, Nucleoside diphosphate kinase; PTPN13, Tyrosine-protein phosphatase non-receptor type 13; RGS3, Regulators of G protein signaling 3; SIK3, SIK family kinase 3; TGF-α, Transforming growth factor alpha; TLR5, Toll-like receptor 5; TOP1, Thimet oligopeptidase 1; UCH, Ubiquitin carboxyl-terminal hydrolase; ZEB1, Zinc finger E-box binding homeobox 1

#### Lung tissues

The total number of identified proteins in lung tissues of dogs in the control, DMVD and DMVD+PH groups were 6,112, 5,260 and 5,433, respectively and the total number of identified proteins were higher than that in the pulmonary arteries. A Venn Diagram revealed that 692 identified proteins were commonly found in the lung tissues of dogs in the control and DMVD groups, while 312 identified proteins were commonly found in the lung tissues of dogs in the DMVD and DMVD+PH groups (Fig 1B). The significant differentially abundant proteins between groups (>2-fold difference, p<0.05) were analyzed and illustrated in a Volcano plot (Fig 2). Out of the 692 identified proteins in lung tissues of dogs in the DMVD group, 76 were found to be differentially expressed compared to the control group (15 upregulated, 58 downregulated) (Fig 2C). Similarly, 15 out of the 312 identified proteins in lung tissues of dogs in the DMVD+PH group were found to be differentially expressed compared to the DMVD group (6 upregulated, 9 downregulated) (Fig 2D).

A set of proteins that were found to have increased or decreased fold-changes in the lung tissues of DMVD dogs compared to the control dogs and in the lung tissues of DMVD dogs with PH compared to those without PH were the focus of this study. In the lung tissues of DMVD dogs compared to control dogs, Histamine N-methyltransferase (HNMT), Catenin delta1 (CTNND1) and Lamin B1 (LB1) were proteins with increased fold-changes, while Thimet oligopeptidase 1 (TOP1), Epithelial splicing regulatory protein 2 (ESRP2) and Intraflagellar transport 88 (IFT88) were proteins with decreased fold-changes. In the lung tissues of DMVD dogs with PH, Toll-like receptor 5 (TLR5), Wnt protein, and Ubiquitin carboxyl-terminal hydrolase (UCH) were upregulated proteins, while Zinc finger E-box binding homeobox 1 (ZEB1), MCF2 cell line derived transforming sequence (MCF2L) and Death associated protein kinase 2 (DAPK2) were downregulated proteins compared to the lung tissues of DMVD dogs without PH (Table 2).

**Fig 3:**
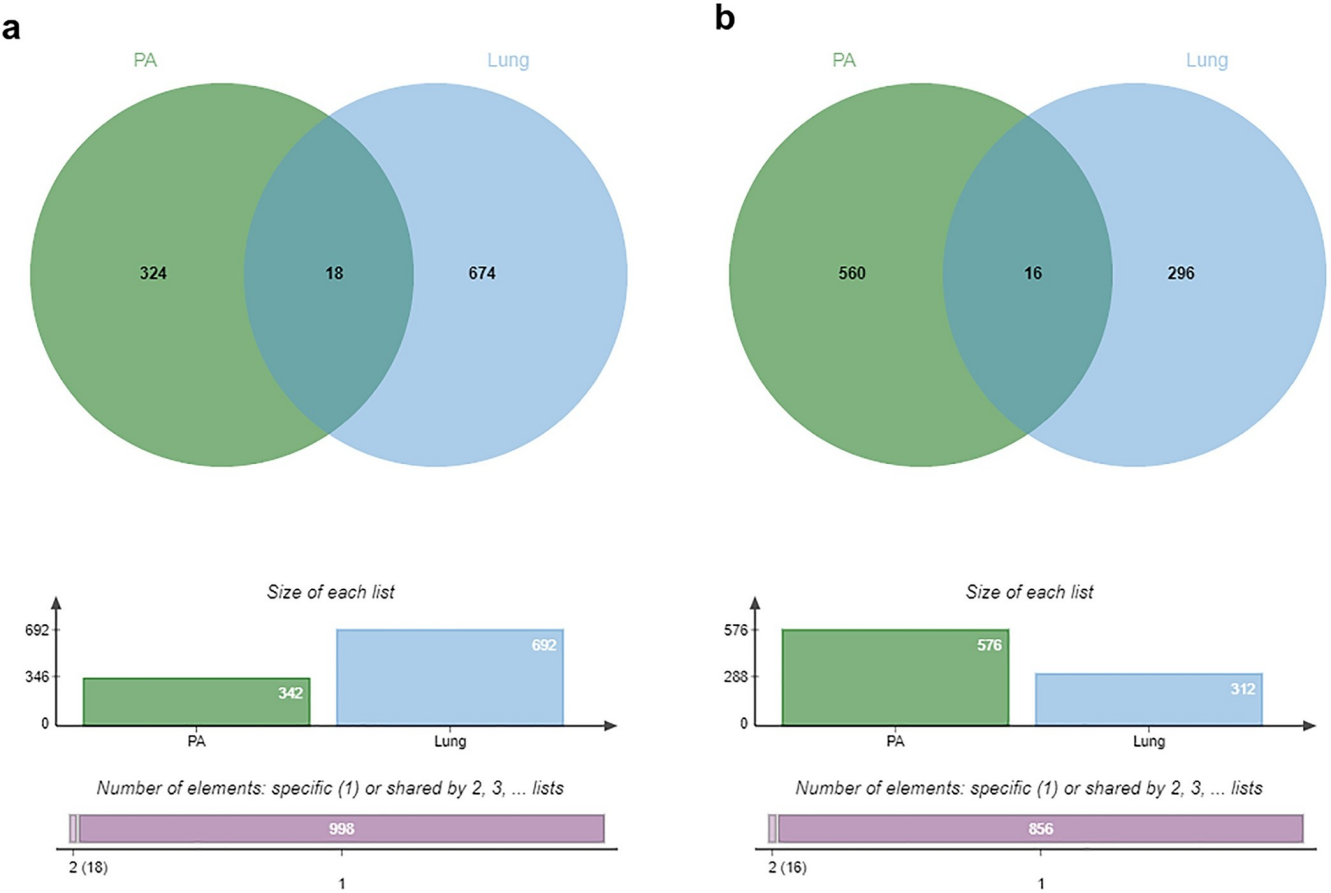
Venn diagram showed the commonly found proteins. Proteins in the control and DMVD groups (a) and the commonly found proteins in the DMVD and DMVD+PH groups (b) that expressed in both pulmonary arteries and lung tissues were displayed as circle. Eighteen commonly found proteins in the control and DMVD groups and 16 commonly found proteins in the DMVD and DMVD+PH groups were expressed in both pulmonary arteries and lung tissues.

Moreover, the commonly found proteins that expressed in both pulmonary arteries and lung tissues were identified, and the results showed that 18 commonly found proteins in the control and DMVD groups (Fig 3A) and 16 commonly found proteins in the DMVD and DMVD+PH groups were expressed in both pulmonary arteries and lung tissues (Fig 3B). According to the differentially expressed proteins, there was only one protein namely insulin-like growth factor II (IGF2) that upregulated in the pulmonary arteries of dogs in the DMVD group compared to the control group was found in both pulmonary arteries and lung tissues. The other differentially expressed proteins were expressed only in the pulmonary arteries or lung tissues.

### The involvement of the differentially expressed proteins and cardiovascular drugs

**Fig 4:**
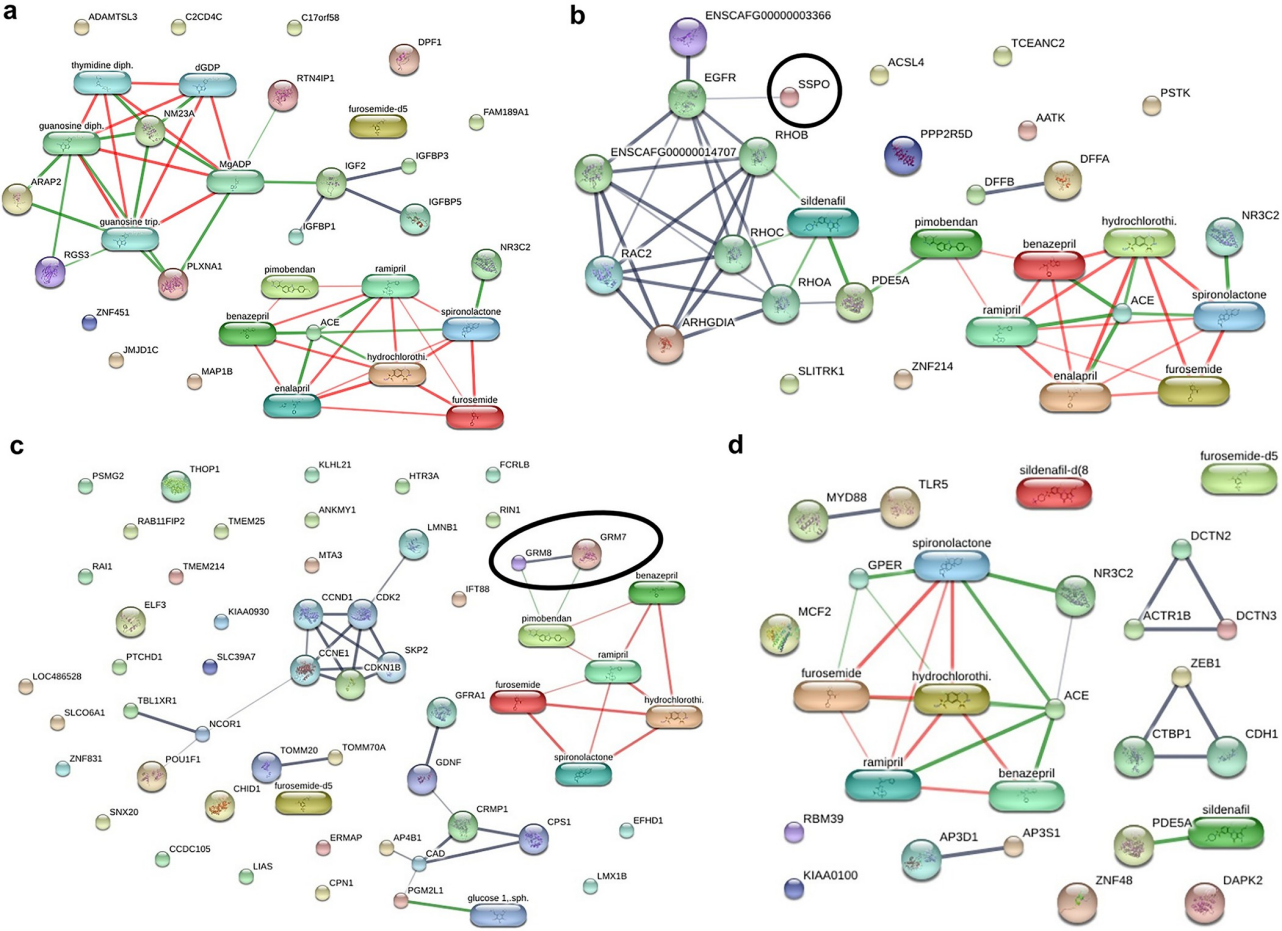
Involvement of the differentially expressed proteins in the networks of proteins- cardiovascular drugs interactions. The edge confidence scores were used to assess the strength of these pathway interactions at functional level. The interactions with high edge confidence scores (>0.700) presenting as thick lines were indicated strong relationships. In the pulmonary arteries, the differentially expressed proteins between the control and DMVD groups were not associated with any common cardiovascular drugs (a), whereas the differentially expressed proteins between the DMVD and DMVD+PH groups including SCO-spondin (SSPO; circled) were weakly associated with sildenafil (b). In the lung tissues, the differentially expressed proteins between the control and DMVD groups showed that Glutamate metabotropic receptor 7 (GRM7) and 8 (GRM8) were associated pimobendan (c) while no differentially expressed proteins between the DMVD and DMVD+PH groups were associated with common cardiovascular drugs (d).

The analysis of proteins-cardiovascular drugs interactions using the STITCH program (version 5.0) found that none of the proteins that were differentially expressed in the pulmonary arteries between the control and DMVD groups were associated with any cardiovascular drugs, as shown in Fig 4A. However, one out of the 29 differentially expressed proteins found in the pulmonary arteries between the DMVD and DMVD+PH groups, specially, SCO-spondin (SSPO), was found to have a weak relationship with the drug sildenafil (Fig 4B). In the lung tissues, the Glutamate metabotropic receptor 7 (GRM7) and 8 (GRM8), which were the differentially expressed proteins in the lung tissues between the control and DMVD groups, were associated with pimobendan (Fig 4C), whereas no differentially expressed proteins in the lung tissues between the DMVD and DMVD+PH groups were found to be associated with any common cardiovascular drugs (Fig 4D).

### Unique expression of proteins identified by LC-MS/MS

**Fig 5:**
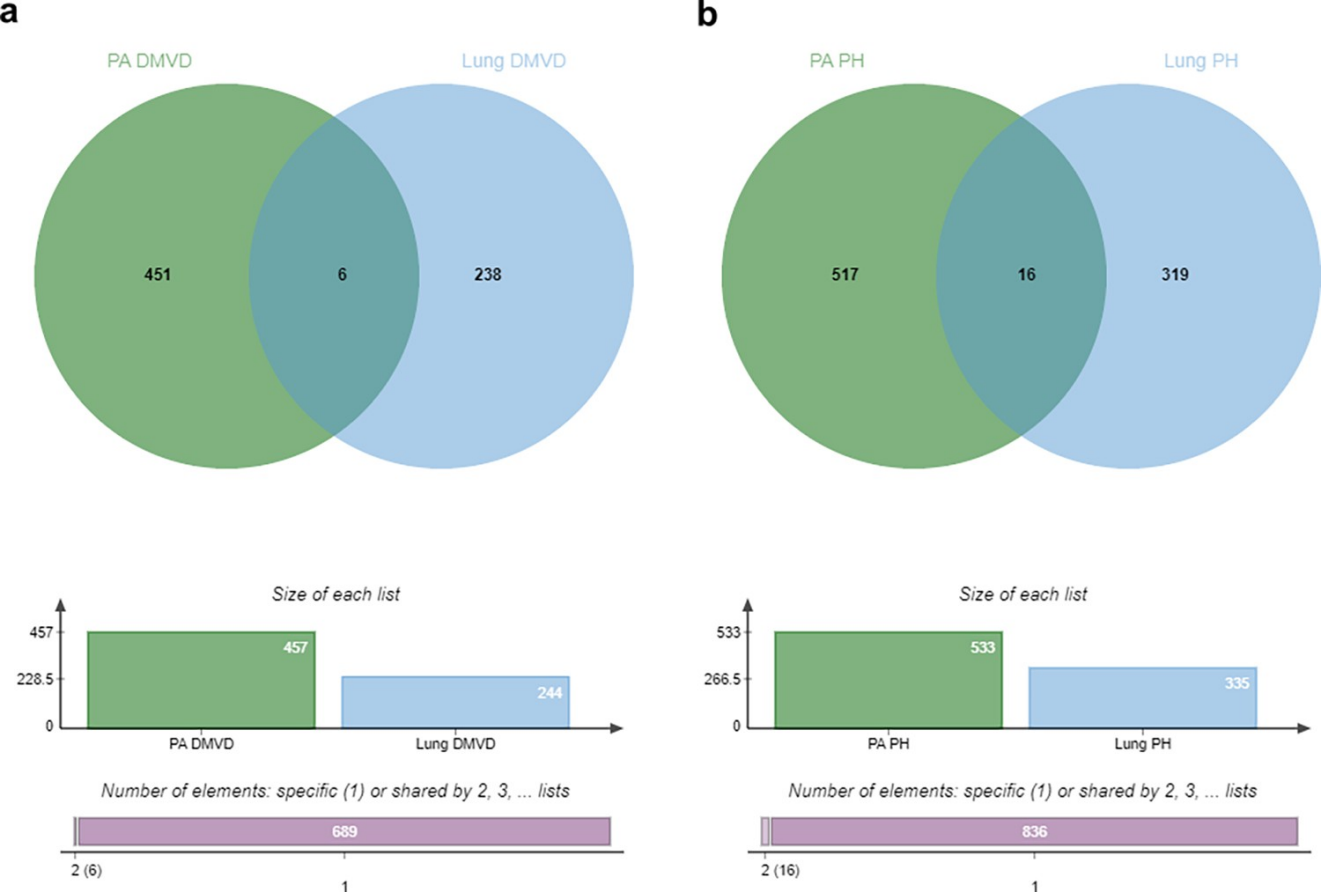
Venn diagram showed the uniquely expressed proteins. Proteins in the pulmonary arteries and lung tissues of dogs in the DMVD groups (a) and the DMVD+PH groups (b). Six uniquely expressed proteins in the DMVD group and 16 uniquely expressed proteins in the DMVD+PH group displayed as circle were expressed in both pulmonary arteries and lung tissues.

In addition to the differentially expressed proteins, the Venn diagram showed that 457 and 533 proteins were uniquely expressed in the pulmonary arteries of dogs in the DMVD and DMVD+PH groups, respectively (Fig 1A). In the lung tissues, 244 and 335 proteins were uniquely found in the DMVD and DMVD+PH groups, respectively (Fig 1B). Upon comparing the unique proteins expressed in the pulmonary arteries and lung tissues, 6 unique proteins were found to be expressed in both pulmonary arteries and lung tissues of dogs in the DMVD group (Fig 5A) and 16 unique proteins were expressed in both pulmonary arteries and lung tissues of dogs in the DMVD+PH group (Fig 5B). Since pulmonary arteries were the main structural changes in PH, this study focused on the unique proteins that are frequently found in the pulmonary arteries of dogs in the DMVD and the DMVD+PH groups considered to be related to the emergence of the medial thickening including Histone H1, Corin and Phospholipase A2 (PLA2) in the DMVD group and Mucolipin-3 (MCOLN3), Neurofibromin 1 (NF1) and Lectin galactoside-binding soluble 3-binding protein (LGALS3BP) in the DMVD+PH group.

## Discussion

The use of LC-MS/MS based proteomic analysis to understand the pathogenesis of PH has been widely studied in human patients and animal models [17]. Many of these proteomic investigations have focused on lung tissues, which are composed of a variety of cell types and may not be the exact location of the pathological alterations in PH. However, previous histopathological studies in dogs have shown that both lung tissues and pulmonary arteries of DMVD dogs, both with and without PH, exhibit changes compared to normal control dogs. Lung tissues of all DMVD dogs, both with and without PH, showed congestion and pulmonary edema, while pulmonary arterial remodeling, especially medial thickening, was present early in DMVD dogs and progressed in DMVD dogs with PH [7, 11].

Proteomics is a high-throughput method that allows for the detection and identification of a large number of proteins in samples without prior knowledge about interested proteins [12]. This study found that thousands of proteins can be detected and identified in the pulmonary arteries and lung tissues of all dogs. As expected, the total number of identified proteins in the lung tissues was greater than in the pulmonary arteries, likely because the lung is a more complex structure than the pulmonary artery [24]. Of all identified proteins in lung tissues and pulmonary arteries, hundreds of proteins were commonly expressed between groups, and several proteins were found to be significant different between groups. The differentially expressed proteins with the largest increased and decreased fold-change between groups were focused on this study to investigate the pathogenesis of PH. This study chose three proteins based on statistical significance and biological relevance to PH. While other identified proteins may be linked to PH, our intentional focus on these top three proteins aims to gain a more in-depth understanding.

Proteomic studies are typically performed on treatment-naïve patients to minimize the impact of medications on the results [16, 25]. However, pulmonary arteries and lung tissues in this study were collected from the dogs with naturally occurring DMVD that were treated with the standard cardiovascular drugs. This may affect the results, but the network of proteins-cardiovascular drug interactions evaluated by the STITCH database showed no association of proteins with the largest fold-changes and cardiovascular drugs, suggesting that the drugs used in this study may not have an impact on the result. Although some differentially expressed proteins exhibited an association with the drugs sildenafil and pimobendan, the confidence score was low, suggesting a less reliable relationship and clinical insignificance [23].

Pulmonary arterial remodeling with medial thickening is a hallmark structural change of PH [2], which also found in DMVD dogs with and without PH [7]. Differentially expressed proteins in the pulmonary arteries of dogs in the DMVD group compared to the control group were identified. These proteins may be related to the pathogenesis of the pulmonary arterial remodeling in DMVD dogs. Upregulated proteins like CACNB3, IGF2 and MAP1B were mainly associated with cell proliferation [26–28] which may play a role in the medial thickening in the DMVD dogs. IGF2 was also found in the lung tissues of DMVD dogs but not significantly different compared to the control dogs. According to IGF2 immunohistochemical staining in lung tissues was detected in smooth muscle cells but not in alveolar cells [29], it was anticipated that IGF2 may be expressed in all sized of pulmonary arteries including small pulmonary arteries in lung tissues. ADAMTSL3 has been reported to be associated with cardiac and vessel formation [30]. NDPK acts as a suppressor by inhibiting cell growth and promoting apoptosis [31]. RGS3, a member of the RGS protein superfamily, has been reported to be expressed in human heart [32] and it was downregulated in failing human heart [33]. The downregulation of proteins such as ADAMTSL3, NDPK and RGS3 in the pulmonary artery of dogs with DMVD may be involved in the pulmonary arterial remodeling seen in this condition. However, further research is needed to fully understand the specific mechanisms and roles of these proteins play in the development of DMVD-associated pulmonary arterial remodeling.

In comparing the expression of proteins in the pulmonary artery of DMVD dogs with and without PH, this study identified differentially upregulated and downregulated proteins. SIK3, one of the members of the salt-inducible kinases family, has been reported to be involved in regulating vascular SMCs proliferation and migration [34]. Collagen type I, is an essential component of the extracellular matrix (ECM) in blood vessels, providing mechanical strength and contractility [35]. An increase in collagen type I is associated with arterial stiffness [36]. TGF-α, is a member of the epidermal growth factor (EGF) family that plays a role in cell proliferation, differentiation, and migration [37]. Overexpression of TGF-α has been shown to induce pulmonary fibrosis and PH in animal models through increased collagen and ECM deposition and increased cellular proliferation. Moreover, when TGF-α expression was inhibited, lung fibrosis and PH partially reversed [38].

AATYK has been found to be necessary for promoting cell differentiation and enhancing apoptosis [39]. HGFA is a serine protease that converts hepatocyte growth factor (HGF) to its active form. HGF induces apoptotic cell death [40]. In animal models of PH, HGF mRNA and protein levels were found to be downregulated in pulmonary artery and lungs. Overexpression of HGF attenuates medial thickening in pulmonary artery [41]. PTPN13, a member of the Class I superfamily of tyrosine-specific phosphatases, has been found to play a role in inhibiting pathways involve in cell proliferation and migration [42]. Downregulation of PTPN13 leads to resistance to apoptosis [43]. Taken together, the upregulation of SIK3, COL1A1 and TGF-α and downregulation of AATYK, HGFA and PTPN13 may contribute to resistance to apoptosis and progression of medial thickening.

The remodeling of pulmonary arteries occurs in both larger and smaller vessels, but studying changes in small pulmonary arteries is difficult because they cannot be easily dissected from lung tissue. In this study, we used peripheral lung tissue as an alternative method to study these changes. However, this approach has limitations because the lung tissue is composed of various structures, not just pulmonary arteries but also other structures. In this study, we found that commonly expressed proteins in lungs were different from those found in pulmonary artery tissues.

HNMT regulates histamine levels in lungs and stimulate airway SMCs contraction [44]. CTNND1 is a protein complex that participated in cell-cell adhesion and promotes cell proliferation and migration [45]. LB1 is a nuclear component involved in regulating of nuclear functions and cell proliferation [46]. TOP1 is a metalloendopeptidase, it hydrolyzes neuroendocrine and cardiovascular peptides [47], and may affect angiotensin converting enzyme II (ACEII) expression in alveolar epithelial cells and vascular endothelial cells that is associated with alveolar fluid clearance [47]. ESRP2 is an RNA binding proteins that controls splicing events during epithelial-mesenchymal transition [48] and is required for maintenance of epithelial cell barriers [49] and lung cell homeostasis [50]. IFT88, a subunit of proteins in the IFT machinery. It regulates ciliary assembly and maintenance and balancing cellular signaling. Expression of IFT88 protein was reported in the cilia of respiratory epithelial cells [51]. Loss of function of IFT88 results in defects in ciliary formation and signal processing [52].

TLR5 is a protein that recognizes bacterial flagellin and acts as an immune sensor in non-immune cells such as epithelial cells and endothelial cells, activating inflammatory response [53] and participating in angiogenic response [54]. TLR5 is highly expressed in airway epithelial cells and endothelial cells during lung infection and vascular regeneration [55, 56]. Wnt is a glycoprotein that activates intracellular signal transduction pathways and has been associated with PH by promoting excessive smooth muscle cell growth [57]. UCH is a subfamily of deubiquitinating enzymes (DUBs) that are involved in cell growth and differentiation, signal transduction, DNA repair, oncogenesis and fibrosis [58]. ZEB1, is a transcription factor that plays a role in the epithelial-mesenchymal transition process and is associated with wound healing and organ fibrosis [59]. MCF2L is a guanine-nucleotide exchange factor that participate in the Rho/Rac signaling pathways and has been reported to be involved with atherosclerosis in humans [60]. DAPK2 is a serine/threonine kinase that participates in several cellular processes including apoptosis, autophagy and inflammation [61]. It is challenging to ascribe the obtained data to the small pulmonary arteries. The upregulated and downregulated proteins in lung tissues may be associated with either lung or small pulmonary artery changes or both in the DMVD and DMVD+PH groups. Further study such as immunohistochemistry should be done to evaluate where these proteins were exactly expressed.

Since the pulmonary artery is the site of structural changes in PH, this study also focused on the proteins that uniquely expressed in the pulmonary arteries of dogs in the DMVD+PH groups, including Mucolipin-3 (MCOLN3), Neurofibromin (NF1) and lectin galactoside-binding soluble 3-binding protein (LGALS3BP).

MCOLN3 is a mucolipin family ion channels that regulates calcium release from endosomes and plays a role in membrane trafficking and fusion in the endosomal pathway [62]. There is limited understanding of the expression and function of MCOLN3 in the pulmonary artery in the context of PH. However, a previous study demonstrated strong expression of MCOLN3 mRNA in the lung tissues of human patients with PH [63]. Neurofibromin 1 (NF1) involved in multiple cellular processes, including cell proliferation, growth, division, survival and migration by participating in various cell signaling pathways. Human patients with pathogenic variants in the NF1 gene have been found to experience pulmonary arterial remodeling and PH. However, the mechanism behind this association remains unknown and requires further investigation [64]. LGALS3BP belongs to the scavenger receptor cysteine-rich domain family and has been found to be involved in immunity and inflammation [65]. As a glycosylated protein, it acts as a ligand for several galectins, such as galectin-3 [66]. Siddaiah et al. (2022) reported a significant increase in LGALS3BP levels in tracheal aspirates from infants with severe bronchopulmonary dysplasia with PH [67].

The first limitation of this study was the absence of the validation step for protein expression, which could have strengthened the reliability of our study. In the past few years, validations of proteomics data have been performed using several methods that require antibodies to detect proteins of interest, such as ELISA, western blot, and immunohistochemistry. Recently, these methods have been suggested as inappropriate for validation because they depend on the specificity of antibodies to detect proteins and the availability of antibodies. Sourcing and developing highly specific antibodies pose major challenges. Moreover, proteomics and other methods prepare samples differently, which might be a significant factor in producing differing results. Therefore, several researchers have ceased conducting protein for current proteomic studies [68–70]. The second limitation of this study was the small sample size. Since this study collected samples from the carcasses of dogs who died naturally, it was challenging to include a sufficient number of healthy old dogs to match the diseased group in the study. Further investigations with a larger sample size should be conducted to strength and support the results of this study.

In conclusion, this study highlights the advantages of using shotgun proteomics to investigate proteins associated with occurrence of PH secondary to DMVD in dogs. The findings of upregulated and downregulated protein in the pulmonary arteries and lung tissues offer valuable insights into the pathogenesis of PH due to DMVD in dogs. These findings may facilitate further study aimed at identifying markers for diagnosis or developing targeted therapeutic methods.
